# Current challenges of research on filamentous fungi in relation to human welfare and a sustainable bio-economy: a white paper

**DOI:** 10.1186/s40694-016-0024-8

**Published:** 2016-08-31

**Authors:** Vera Meyer, Mikael R. Andersen, Axel A. Brakhage, Gerhard H. Braus, Mark X. Caddick, Timothy C. Cairns, Ronald P. de Vries, Thomas Haarmann, Kim Hansen, Christiane Hertz-Fowler, Sven Krappmann, Uffe H. Mortensen, Miguel A. Peñalva, Arthur F. J. Ram, Ritchie M. Head

**Affiliations:** 1 Department of Applied and Molecular Microbiology, Institute of Biotechnology, Berlin University of Technology, Gustav-Meyer-Allee 25, 13355 Berlin, Germany; 2 Department of Systems Biology, Technical University of Denmark, Building 223, 2800 Lyngby, Denmark; 3 Department of Molecular and Applied Microbiology, Leibniz Institute for Natural Product Research and Infection Biology, Hans Knöll Institute (HKI), Jena, Germany; 4 Department of Microbiology and Molecular Biology, Institute of Microbiology, Friedrich Schiller University, Jena, Germany; 5 Department of Molecular Microbiology and Genetics, Institute of Microbiology and Genetics, Georg-August-Universität Göttingen, Grisebachstr. 8, 37077 Göttingen, Germany; 6 Institute of Integrative Biology, University of Liverpool, Biosciences Building, Crown Street, Liverpool, L69 7ZB UK; 7 Fungal Physiology, CBS-KNAW Fungal Biodiversity Centre and Fungal Molecular Physiology, Utrecht University, Uppsalalaan 8, 3584 CT Utrecht, The Netherlands; 8 AB Enzymes GmbH, Feldbergstr. 78, 64293 Darmstadt, Germany; 9 Biotechnology Research, Production Strain Technology, Novozymes A/S, Krogshoejvej 36, 2880 Bagsvaerd, Denmark; 10 Mikrobiologisches Institut – Klinische Mikrobiologie, Immunologie und Hygiene, Friedrich-Alexander University Erlangen-Nürnberg and University Hospital Erlangen, Wasserturmstr. 3/5, 91054 Erlangen, Germany; 11 Department of Cellular and Molecular Biology, Centro de Investigaciones Biológicas CSIC, Ramiro de Maeztu 9, 28040 Madrid, Spain; 12 Molecular Microbiology and Biotechnology, Institute of Biology Leiden, Leiden University, Sylviusweg 72, 2333 BE Leiden, The Netherlands; 13 Ceratium Limited, The Haven, West Kirby, CH48 8AP UK

## Abstract

The EUROFUNG network is a virtual centre of multidisciplinary expertise in the field of fungal biotechnology. The first academic-industry Think Tank was hosted by EUROFUNG to summarise the state of the art and future challenges in fungal biology and biotechnology in the coming decade. Currently, fungal cell factories are important for bulk manufacturing of organic acids, proteins, enzymes, secondary metabolites and active pharmaceutical ingredients in white and red biotechnology. In contrast, fungal pathogens of humans kill more people than malaria or tuberculosis. Fungi are significantly impacting on global food security, damaging global crop production, causing disease in domesticated animals, and spoiling an estimated 10 % of harvested crops. A number of challenges now need to be addressed to improve our strategies to control fungal pathogenicity and to optimise the use of fungi as sources for novel compounds and as cell factories for large scale manufacture of bio-based products. This white paper reports on the discussions of the Think Tank meeting and the suggestions made for moving fungal bio(techno)logy forward.

## Executive summary

### The EUROFUNG network

The EUROFUNG network currently includes 35 academic members and an industrial platform of 9 European biotechnological and pharmaceutical companies, representing a multidisciplinary virtual centre in the field of fungal biotechnology. The mission of EUROFUNG is to catalyze new research activities and collaborations across disciplines and institutions, to act as a vehicle for communication, and to raise awareness amongst stakeholders of priority areas to advance fungal technologies.

### The importance of fungi for human welfare and the bioeconomy

European companies such as AB Enzymes, BASF, Bayer, Chr. Hansen, DSM, DuPont, Novozymes, Puratos and Roal Oy are global leaders in using filamentous fungi as cell factories in white and red biotechnology. This group of microorganisms is often superior to bacterial and yeast based production systems, in terms of metabolic versatility, robustness and secretory capacity. Large-scale manufacturing processes have been developed for the production of organic acids, proteins, enzymes and small molecule drugs including antibiotics, statins and steroids. Fungal biotechnology thus plays a central role for many industries including food and feed, pharma, paper and pulp, detergent, textile and bio-fuel. For example, the worldwide production of citric acid produced using the filamentous fungus *Aspergillus niger* by far exceeds the production of any other organic acid made by microbial fermentation. Indeed, the production of plant biomass degrading enzymes by filamentous fungi alone is a €4.7 billion market, which is expected to double in the next 10 years. However, filamentous fungi also pose a serious threat in relation to food supply and safety as they reduce worldwide annual crop yields by at least 10 %, with the emergence of new hypervirulent isolates and the spread of pathogens to new geographic areas continually reported. Accordingly, the global demand for fungicides to control fungal diseases was a €10 billion market in 2014 and is expected to grow continuously in the next decade. However, increased agricultural application of fungicides in recent years has also led to the development of resistance in fungal pathogens that cause diseases in the clinic such as pulmonary Aspergillosis. These fungal infections threaten the health of more than 1.2 billion individuals worldwide and claim the lives of about 1.5–2 million people every year.

### Gaps in knowledge and technologies and strategies to address these challenges

To better fight and sustainably exploit filamentous fungi, research and development has to significantly advance on three different levels:

(i) *Science*: to overcome our limited understanding of fungal biology, the lack of sustainable use and efficient integration of post-genomic data, as well as insufficient availability of synthetic biology tools
(ii) *Community*: to strengthen and connect a rather fragmented community consisting of basic researchers, medical mycologists, plant pathologists and industrial microbiologists by increased funding for multidisciplinary, collaborative and translational projects
(iii) *Personnel*: to provide interdisciplinary training for young scientists and technicians that goes beyond narrow sub-disciplines

Table 1 summarises ten research opportunities with the highest priority identified during the Think Tank meeting and the most pressing resources required to address these. Further research opportunities, in addition to those highlighted below will be discussed in detail in the main text of the white paper including respective recommendations on how to achieve them.

**Table 1:** Summary of the EUROFUNG’s Think Tank

Research opportunities	Community resources required
Understanding basic principles underlying fungal growth, development and gene expression	Investment in a well-structured dedicated science base including European and international initiatives (e.g. COST actions) to optimise knowledge exchange and efficient multidisciplinary activities
Development of publicly available gene deletion and overexpression mutant libraries	Sustainable investment in appropriate strain culture collections
Development of high-throughput technologies for cloning, mining and screening of fungal strains	Combined activity between technology developers and experts in fungal science to modify technologies according to specific challenges of filamentous fungi
Development of genome editing and synthetic biology tools for high-throughput manipulation and (re)design of fungal genomes	Dedicated funding for methods and tool development regarding generic synthetic parts and easily adaptable molecular components for integration into experimental pipelines for filamentous fungi
Development of publicly available, high-quality manually curated omics databases where fungal genomes and omics-studies can be shared	Continuous and suitable investment in appropriate bioinformatics resources, structures and staff training
Development of strains with improved protein expression and secretion rates	
Development of strains with improved macroscopic morphologies in submerged cultures	
Development of optimal/minimal genomes lacking unwanted mycotoxin genes	
Understanding fungal pathogenesis in different hosts and across kingdoms including the development of infection models	Dedicated research activities including cross sector forums to investigate human, animal and plant pathogens and disease ecology. Direct links to clinical and agricultural global science activities and policy makers (WHO, FAO, EC)
Selection of appropriate targets for antifungals and establishment of drug development strategies	Dedicated funding for screening programmes to identify new modes of action against key targets and translation into well-financed early stage development pipelines

## Background

The knowledge-based bio-economy is critical for European growth and development and has been prioritized by the EC as a key area that will underpin long term sustainable growth [1]. More specifically, Europe is a world leader in the industrial applications of filamentous fungi and the underlying science. This group of organisms currently has a major commercial impact in product areas including food and feed, pulp and paper, textiles, detergents, beverages, bio-fuel and chemical production, food spoilage and disease. With over 6 million species [2], fungi represent a major resource for future sustainable applications and processes, but also a potential foe, since pathogenic fungi are a serious and growing cause of human disease and mortality and a threat to crops and food security. Consequently, fundamental and applied research dedicated to this broad group of organisms is both important and timely.

A Think Tank was organised by the EUROFUNG network on April 28–29, 2016, in Berlin, Germany to discuss current and future research opportunities and future challenges in fungal biology and biotechnology over the next 10 years. Industrial and academic contributors defined the tools, technologies, infrastructures and resources required to identify and address challenges, and advance fungal biotechnology as a key contributor to the European bioeconomy that is essential to sustainable growth. Founded in 1995, the EUROFUNG network currently includes 35 academic members and an industrial platform of 9 European biotechnological and pharmaceutical companies (http://mikrobiologie.eurofung.tu-berlin.de/). This represents a European virtual centre of multidisciplinary expertise with globally recognised experts and long-term industry/academia collaborations, specifically in the field of white biotechnology and fungal biology in general. The EUROFUNG mission is to catalyze new research activities and collaborations, to act as a vehicle for communication and exchanges between academia and industry, and to raise awareness amongst key policy makers, funders and the broader stakeholder’s community of priority areas to advance fungal technologies. This white paper summarizes the discussions on gaps in knowledge and technologies and the strategies to address these challenges.

## The importance of filamentous fungi

The global sale volume of white biotechnology products was estimated to be €110 billion in 2008 which is expected to increase fourfold to €450 billion by 2020 [3]. The proportion of chemicals produced by biotechnology will increase significantly within a generation, with biotechnology products expected to dominate the speciality chemicals sector by 2030. European companies such as AB Enzymes, BASF, Bayer, Chr. Hansen, DSM, DuPont, Novozymes, Puratos and Roal Oy are global leaders in using fungi for bulk manufacturing of organic acids, proteins, enzymes and secondary metabolites. The worldwide production of citric acid produced with the filamentous fungus *Aspergillus niger* is about 1.6 million metric tons, a number that far exceeds the production of any other organic acid made by fermentation [4]. It is the most widely used organic acid in foods, beverages, pharmaceuticals and technical applications and the market size continues to grow. Itaconic acid produced by *Aspergillus terreus* is a commodity used by the chemical industry for the production of synthetic resins, fibres, plastics, rubbers, surfactants, and oil additives, which has resulted in an increased demand for this product. Other multifunctional organic acids of filamentous fungal origin such as succinic, fumaric and malic acid are exploited as commodities for the manufacture of biodegradable polymers. Fungal enzymes are used within large-scale manufacturing processes, including paper and pulp, food and feed, beverages, wine, detergents, textiles, and bio-fuel production. Large-scale production of key building blocks for active pharmaceutical ingredients has also been successfully established in filamentous fungi as exemplified by Bayer’s steroidal progrestin drospirenone (>€0.54 billion in the first 9 months of 2015). Indeed, the majority of enzymes used worldwide are produced by filamentous fungi. In 2015, the global market for industrial enzymes was estimated to be worth 3.5 billion euros, with Novozymes and Dupont having a 48 and 20 % share of the market respectively [5]. Notably, this industry is engaging interest from other agricultural suppliers, including Bayer CropScience, BASF, Syngenta/DSM, and Chr. Hansen [5]. Several species of *Aspergillus* as well as other fungi, including *Trichoderma reesei* and *Myceliophthora thermophila,* are important for industrial enzyme production. The metabolic diversity of fungi and the broad range of ecological niches they inhabit mean that many species, especially the basidiomycetes, have significant potential as sources of novel enzymes for future exploitation.

Fungal bioactive compounds ranging from antibiotics to statins have saved the lives of millions since the discovery of penicillin by Alexander Fleming in the 1920s. These are generally secondary metabolites or their derivatives and their industrial production is and will continue to be a major activity. Again there is significant potential in the identification and production of novel compounds and to re-design and repurpose them by means of synthetic biology. However, antimicrobial resistance of bacteria is currently spreading, whereas the number of newly discovered antibiotics is declining [6]. The dramatic increase in antimicrobial resistance has led the World Health Organization to call for urgent and concerted action on research and innovation to develop novel tools [7] and the G7 health ministers to call for accelerated research and production of new antimicrobial agents (Declaration of the G7 Health Ministers, Summit 2015).

Fungal pathogens kill more people per year globally than malaria or tuberculosis (estimated 1.6–2 million [8]). The main causative agents of human deaths are found in the genera *Cryptococcus, Candida,* and *Aspergillus*, whereby the genus *Aspergillus* causes between 38 and 80 % of fungal disease-associated mortality across Europe, depending on geographical location [9]. The damage caused by fungi to global cereal production including rice, wheat and maize is also alarming. More than 10 % of the annual harvested crops are estimated to be spoiled by fungi [10] and the global demand for fungicides was a €10 billion market in 2014 and expected to grow at 5.3 % by 2019 [11]. Finally, there is an important and increasing need to detect fungi and their metabolites in relation to food safety and security, crop protection, domestic environments (i.e. as a cause of sick building syndrome), and clinical diagnosis, as recognized through recent EU legislation.

## Key challenges to exploit and fight filamentous fungi

The Think Tank identified several key barriers which limit optimum exploitation of filamentous fungi and hamper our ability to design new and sustainable antifungal strategies. The barriers fall into three categories:

(i) Science: The limited range of molecular and synthetic biology tools and high-throughput technologies tailored to filamentous fungi delays research, product development and the identification of antifungal targets, limits flexibility in engineering the required combinations of genome modifications and appropriately regulated transgenes. Effective integration of the rapidly growing post-genomic data is limiting systems biology, while also hampering metabolic modelling and engineering approaches. In particular, advanced metabolomic analysis and profiling of fungal organisms is not well developed in spite of the obvious potential for both exploitation and manipulation of their diverse metabolism. Overcoming these limitations will facilitate the development of new products and biosynthetic systems. Finally, fungal biotech companies are dependent on poorly defined, highly mutagenized, production strains. This can be overcome by the rational development of strains with optimum genomes, providing defined robust hosts for synthetic biology and accelerated product development.
(ii) Community: The range of organisms and the diversity of potential products associated with filamentous fungi is reflected in a community which is inevitably broad but also fragmented. One consequence is the limited number of appropriate EU calls or multinational collaborative projects in spite of significant funding from national programs. Increased European funding would help reduce fragmentation and better explore this diversity and potential.
(iii) Personnel: The long-term development of fungal biotechnology is dependent on recruiting the best young scientists and research staff, providing them with effective, interdisciplinary training that goes beyond narrow sub-disciplines, giving them the confidence, enthusiasm and networks to progress in their careers.

The participants agreed that the coming decade will offer many opportunities and challenges for R&D on filamentous fungi. The exploitation of filamentous fungi will contribute significantly to the realisation of a secure, sustainable, bio-based future. To achieve this, the following key questions and challenges need to be answered:

1. How do we exploit filamentous fungi in more efficient and sustainable ways in biotechnological, pharmaceutical and agrochemical industries?
2. What is the best way to develop new antifungals and sustainable strategies to fight plant and human pathogens?
3. How can synthetic biology contribute to the design of optimised fungal genomes?
4. What are the optimal approaches to analyse and provide access to fungal–omics data to realise their full potential?
5. What are fungal model organisms good for?
6. Which technologies need to become developed and integrated to better understand fungal growth and development?
7. Which training programs are key for the next-generation of fungal scientists?

## Exploitation of filamentous fungi by biotechnological, pharmaceutical and agrochemical industries

Historically, filamentous fungi were used to produce enzyme complexes containing a number of different activities necessary for degradation of e.g. plant materials like (ligno-)cellulose, starch, pectin, hemicellulose, protein, and lipids. These enzyme activities are still widely produced but the products today are more refined. Traditional strain improvements were mainly achieved by mutagenesis and screening to remove specific unwanted activities and for general increase of expression of complex components. The advent of recombinant technologies enabled tailoring of enzyme complexes by specific deletions and overexpression became possible. Development of clean production hosts deleted for unwanted extracellular proteins also improved product quality and production economy. Further, optimized variants of natural enzymes have been enabled by protein engineering e.g. to improve stability at relevant industrial conditions (temperature, pH). Today, the main enzyme activities produced in fungi are used in the food industry (alpha-amylases, glucoamylases, proteases, pectinases, xylanases), the feed industry (phytases, xylanases), the biofuel industry (glucoamylases, invertases, (hemi)-cellulases) and the detergent industry (lipases, endo-glucanases). However, achievable expression levels of similar proteins still varies a lot and some enzymes are very difficult to produce in a cost-effective manner, so more versatile and higher yielding host strains are needed to extend the range of commercial enzymes.

Several decades ago, fungal microorganisms enabled oxidative functionalization of natural steroids (e.g. Upjohn’s cortisone process [12]) and currently, they are used in the production of steroidal drugs and oxidative functionalization of steroidal [13] and non-steroidal building blocks. There is a renewed and growing interest of pharma industry in using filamentous fungal enzymes, particularly for catalysing biooxidations. Since 75 % of drugs are metabolised by human p450 cytochromes [14], fungal oxygenases are of interest to mimic human liver biotransformations. In addition, oxidative functionalization of natural products or bioactive leads is an emerging technology for providing novel chemical entities to bioactivity testing. Fungi are a rich source for highly relevant biooxidation catalysts; but know-how about specific monooxygenases and their coupled oxidoreductases is very limited and precludes further optimization options including directed evolution approaches for turnover or selectivity fine tuning. However, combining fungal monooxygenases with fungal or non-fungal host systems for developing a fit-for-purpose biooxidation toolbox may allow for production-scale application of enabling CH-biooxidation processes overcoming current limitations of recombinant *E. coli* or yeast based biooxidation catalysts. Furthermore, transaminases, imine reductases, ketoreductases and lipases are the most relevant enabling enzyme classes for pharma R&D applications. Using filamentous fungi for transaminases as well as imine and keto reductases would be highly attractive synthetic chemists in pharma R&D if benchmarking against currently available enzymes shows a broadened substrate scope or beneficial stability profiles.

Finally, natural products still play a role in pharma R&D for identifying novel modes of actions or as early leads. Hence, novel natural products (e.g. from the hidden treasures of fungal genomes) should be screened and profiled in biological systems with a clear need for new mode-of-actions (e.g. activity, modulation of tumor immune response, inhibitors for chromatin modifying enzymes). For cancer indications, novel natural products may be interesting either as antibody drug-conjugates payloads or if they show unprecedented antiproliferative fingerprints (e.g. selective killing of cell lines from specific tumor indications). As synthetic access is still the major limitation for using natural products as pharma leads, synthetic biology approaches are highly attractive to overcome this hurdle. With a capacity to produce several dozens of secondary metabolites per filamentous fungus and an estimated diversity of 6 million fungal species [2], there are potentially millions of novel bioactive compounds awaiting their discovery. To unlock this treasure chest (often referred to as ‘biosynthetic dark matter’), the development of new bioinformatics technologies is required that mines (meta)genomic data and connect them with metabolomics and phenotypic data [15, 16].

### Filamentous fungi as production hosts

Industrially used filamentous fungi are often superior to bacterial and yeast based production systems, in terms of versatility and secretory capacity, but to date only a small fraction of the potential of filamentous fungi has been exploited or is even known. The main reasons are: (i) the physiology behind secretion is still poorly understood, (ii) knowledge on improved protein secretion for one protein is not necessarily transferable to other proteins or fungal systems, (iii) limitation of productivity due to non-optimal macroscopic morphologies and (iv) unwanted mycotoxin production during fermentation.

The key challenge is to maximise production at minimal cost, to ensure robustness and accelerate the identification, engineering and development of new products. There are seemingly three strategies going forward other than just carrying on as we are, gradually optimizing existing production systems: (1) rationally design new hosts using synthetic biology, (2) specifically introduce beneficial changes identified in established production strains and (3) edit out problematic components of fungal genomes, e.g. mycotoxin encoding genes, developmental genes or unwanted proteases. The participants agreed that the following developments are of general importance for filamentous fungal cell factories:

- high-throughput technologies for submerged, surface growth and co-cultivations
- development of production strains with regulated developmental stages to optimize growth in industrial conditions
- high-throughput technologies for cloning, mining, expression and screening mutants
- high-throughput technologies for strain constructions
- annotation tools for in silico selection of promising enzymes and therapeutic targets from genomics data
- robust computational tools for protein engineering (e.g. using machine learning) to fit enzymes better to industrial applications
- miniaturization of cultivation using microtiter plates and optimization of microbioreactors
- development of single-cell analytic tools to study the stochastic nature of gene expression and protein synthesis
- development of production strains with no or reduced harmful secondary metabolites production including mycotoxins
- development of production strains that detoxify mycotoxin-contaminated raw materials
- better process designs and process controls for improved production

### Fundamental questions regarding fungal exploitation remain a key challenge

Although there is such a long track record in exploiting filamentous fungi as cell factories, our current knowledge and understanding of fundamental cellular and developmental processes is severely limited, with many key questions unanswered. Why are many filamentous fungi not able to grow in submerged culture? What are the triggers and signals perceived by fungi when they grow on surfaces or in liquid media? Why are some isolates good producers on solids but not liquids? How can surface production be reverse engineered into liquid growth? Why and how does morphology adapts to different substrates and environmental conditions? What defines optimal gene structure for expression? Paradoxically, successful protein and process engineering has resulted in many more ‘black boxes’. Protein improvements are non-generic and improvements for protein A production are not necessarily transferable to protein B or C.

Crucially, the basis of many of the changes underlying strain improvement are either undefined or not in the public domain. Insights into the genomic evolution of production strains which underwent decades of rational and non-rational strain improvement programs have, due to commercial confidentiality, not been made available to academia. Hence, industry has become repositories of key knowledge, which has the potential to transform our fundamental understanding of fungal metabolism, growth and development and that could underpin the use of synthetic biology to engineer robust strains and speed product development. Exploring mechanisms and collaborations to realize this potential will be challenging but should represent a priority for the whole community.

Finally, a number of fungi also establish symbiotic relations with plants forming mycorrhizal structures with their hosts. These mutualistic relationships are not only fundamental for the cycling of carbon and nitrogen in the environment, but also contributes to protect the plant hosts against pathogens [17, 18].

## The urgent need to fight human- and phyto-pathogenic fungi

Ensuring global food security represents one of the greatest challenges facing humanity in the 21st century. The pressures on food production are immediate, but by 2050 the world will need to increase agricultural production by at least 30 % in order to feed its anticipated population of 9 billion [19]. A multitude of fungal pathogens cause diverse diseases like rusts, smuts, blasts, blotches and mildews of staple crops (Table 2), which ultimately destroy enough food annually to feed 600 million people, approximately 8.5 % of the total population [20]. Fungal disease outbreaks also threaten species that indirectly maintain crop health, such as the ascomycete *Pseudogymnoascus destructans*, which causes catastrophic epidemics of bats and a subsequent increase in crop-destroying insects [21]. With regards to climate change, trees that are lost or damaged by fungi fail to absorb 230–580 megatonnes of atmospheric CO_2_ [20].

**Table 2:** Top 10 fungal pathogens of plants [24]

Rank	Fungal pathogen	Affected plant(s)	Name
1	*Pyricularia oryzae*	Rice and wheat	Rice blast
2	*Botrytis cinerea*	>200	Grey mould
3	*Puccinia* spp.	Wheat	Rust
4	*Fusarium graminearum*	All cereals	Head blight
5	*Fusarium oxysporum*	Multiple	Vascular wilt
6	*Blumeria graminis*	Grasses	Powdery mildew
7	*Zymoseptoria tritici*	Wheat	Septoria blotch
8	*Colletotrichum* spp.	All crops	Spots and blights
9	*Ustilago maydis*	Corn	Corn smut
10	*Melampsora lini*	Flax	Flax rust

Worryingly, the battle lines between fungal pathogens and plants are constantly expanding, with the emergence of new hypervirulent isolates and the spread of pathogens to new geographic areas. As just one example, the devastating wheat blast disease caused by *Magnaporthe oryzae* was recently reported in Bangladesh [22]. This agricultural emergency is the first case of this disease in Asia and has serious implications for wheat production throughout the continent. Pathogen geographical expansion is likely facilitated by global warming [23], and of extreme concern is emergence of multi fungicide resistant crop-destroying fungi. Given the serious and expanding threats fungi pose to crop and plant health, the EUROFUNG consortium advocates that improved pathogen biosecurity protocols, new fungicides, and disease resistant plants are urgent scientific and political goals.

With regards to infectious diseases of humans, it is likely that agricultural application of fungicides has also led to the development of resistance in fungal pathogens that cause diseases in the clinic, such as the major pulmonary pathogen *Aspergillus fumigatus* [25]. Affecting more than 1.2 billion individuals worldwide and killing about 1.5–2 million of them, fungal pathogens pose a serious global threat [8]. The main causative agents of human deaths after opportunistic fungal infections are found in the genera *Cryptococcus*, *Candida*, and *Aspergillus* (Table 3, from [26]), whereby the latter is linked to 30–95 % of fungal disease-associated mortality. Even with treatment, mortality of invasive aspergillosis remains high and increases to 90 % in cases with drug resistant isolates [27]. Besides opportunistic infections caused by fungal pathogens that mainly affect immuno-compromised patients, primary pathogenic fungi can cause severe diseases but are mainly restricted to endemic areas [26]. It is the steadily increasing group of immunosuppressed patients at risk for fungal infections, resulting in severe complications in treating the underlying disease condition. This is not only associated with significant mortality rates but also increasing costs of treatment [28]. Given that disease is the outcome of a complex pathogen-host interplay, understanding the mechanisms of both immunity as well as of fungal virulence is key in developing innovative therapeutic strategies.

**Table 3:** Most common diseases caused by fungal pathogens infecting immunosuppressed individuals [26]

Disease	Cases/year	Mortality rates (%)
Aspergillosis	>200,000	30–95
Candidiasis	>400,000	46–75
Cryptococcosis	>1,000,000	20–70
Mucormycosis	>10,000	30–90
Pneumocystis pneumonia	>400,000	20–80

### The fungicide and antifungal pipeline is running dry

Fungicides are a critical weapon in the arsenal to protect crop yields. There are 12 fungicide mode of actions, including the inhibition of nucleotide synthesis, respiration, and signal transduction, among others [29]. These can be further divided into target sites for which chemical groups are active. The most common modes of action are targeted by compounds that inhibit respiration or sterol biosynthesis (23 % and 22 % of compounds respectively [29]). Molecules with new modes of action are urgently required due to widespread pathogen resistance to many fungicides. For example, the wheat pathogen *Zymoseptoria tritici* has growing resistance to demethylation inhibitors [30], and recent reports have identified highly resistant isolates to the other main control fungicides, succinate dehydrogenase inhibitors [31]. Worryingly, the number of new active ingredients introduced and in development worldwide to combat *Z. tritici* has fallen over the last 30 years [30]. There are similar limitations for the fungicide discovery pipeline for all pathogens of major crops [29]. The participants unanimously agreed that the development of the next generation of so called ‘smart’ fungicides is an urgent priority: highly active, broad spectrum, low dose, environmentally safe compounds.

For human-pathogenic fungi, a clear lack of novel targets for antifungal substances is evident too [8]. Currently approved drugs interfere with the fungal plasma membrane, ergosterol biosynthesis, cell wall biosynthesis, or nuclear function as main modes of action. Novel compounds that may act over a broad spectrum of species are not in sight, which is alarming given the emergence of resistance against some of the most established therapeutics. All this is exacerbated due to a chronic lack of investment in the antifungal pipeline accompanied by challenges in developing such substances when dealing with a eukaryotic pathogen.

### A roadmap towards new antifungal strategies

Advances in fundamental science, and the emergence of fungal omics datasets, have arguably not been harnessed by organizations that coordinate global plant biosecurity. There are currently nine inter-governmental plant protection organizations, including the European and Mediterranean Plant Protection Organization (EPPO). These organizations propose protocols for regional phytosanitary measures and run databases for disease recognition, among many other roles. A key theme identified by the EUROFUNG consortium was the rapid expansion of fungal omics data, and their curation in publicly available online compendia. These advances have been largely ignored in the design and implementation of biosecurity protocols, where risk assessments are based on lists of known, well characterized fungal pathogens of plants. As noted by others, such taxon based approaches ignore threats from the many undescribed fungal pathogens, do not adequately consider emergence of hyper virulent lineages, and are vulnerable to threat mismanagement due to highly complex definitions of fungal species [32]. Consequently, advances in fungal omics technology and databases, most obviously comparative genomics, will enable biosecurity protocols to be implemented based on systems level biology. Integration of omics compendia with existing biosecurity alert databases (e.g. the EPPO Global Database, https://gd.eppo.int/) might be one way of addressing current shortcomings in biosecurity measures.

With regards to development of smart fungicides, improved fundamental understanding of pathogen biology, at individual gene, protein and systems levels, will enable the next generation of compounds to be delivered. Encouragingly, there have been many recent advances in genetic, molecular and cellular tools that enable the multifaceted infection phenotype to be dissected in unparalleled detail [33]. These include robotic screening and automated analysis protocols, improved virulence assays, mutant isolates with elevated rates of homologous recombination for efficient targeting of DNA cassettes in recipient genomes, heterologous expression systems, recyclable markers, and, most recently, CRISPR/Cas9 genome editing (see below). These tools and techniques have identified new potential pathogen targets, based on essentiality for disease and other promising criteria for rational chemical inhibition. These might include, for example, identification of putative targets in the cell wall, a fungal specific structure which obviates the need for compound transition across the cell membrane. Alternatively, rational targets include processes that are prerequisites for disease initiation, when pathogen abundance is low and plant damage yet to occur. It is therefore important to prioritize methodological initiatives that develop tools and resources for the diverse range of crop pathogens (Table 1). Most notably, these should include CRISPR/Cas9 genome editing, a technique that offers unparalleled potential for accurate, versatile and high throughput manipulations of fungal genomes [34, 35]. Concomitantly, the EUROFUNG consortium advocates the development of publicly available pathogen deletion or over-expression libraries, which avoids laborious cloning for research groups, and enables functional genomic analyses of hundreds or thousands of genes in parallel [36]. Although expanding the repertoire of rational pathogen targets is a vital component of the fungicide discovery pipeline, determining which of these are able to be inhibited by a compound—finding so called ‘drugable’ targets—is a highly challenging. Obviously, compound libraries are a critical resource for overcoming this bottleneck, yet access to large, biologically active, structurally diverse compound libraries is limited for research groups outside industry. Indeed, it is clear that the capabilities of industry to conduct and interpret chemical screens are far more advanced that most non-industrial counterparts; Syngenta, for example, are able to screen 300,000 compounds per year [30]. Moreover, industry has the necessary expertise and infrastructure to develop new chemical leads and ultimately bring novel fungicides to market. Consequently, private–public partnerships hold huge potential for translating new pathogen targets or chemical leads into the next generation of smart fungicides.

For developing novel agents acting against human-pathogenic fungi, several deficiencies in research are evident, among them the lack of comprehensive mutant libraries and shortcomings in infections models. In modeling the host-pathogen interactions that result in disease, systems biology has to be seen as state-of-the-art approach. Within this field, network analyses can be applied to predict common points of attack that may be exploited for future antifungal therapies against a wide range of human-pathogenic fungi. Such network modeling approaches can comprise regulatory circuits or fundamental aspects of fungal physiology, like primary metabolism [37]. A crucial prerequisite for addressing putative virulence determinants in fungal pathogens is an advanced molecular biology that allows precise and defined manipulation of the fungal genome of interest and tight conditional gene expression. In this respect, several limitations and differences exist among the range of human-pathogenic fungi, hampering the identification of general principles of fungal virulence. To address this requires the implementation of smart tools for fungal molecular biology accompanied by the generation of genome-wide mutant strain libraries. This will allow the development of efficient screens for antifungal targets such as essential genes. These may then be analyzed with respect to their drugability to provide perspectives for novel and innovative antifungals with new modes of action. The fact that most antifungals are fungistatic rather than fungicidal requires special attention with respect to the emergence of resistance and secondary effects. Furthermore, the usual timelines for development and approval, which is about 10 to 15 years, underlines the need for urgent action and acceleration of the processes that define the antifungal pipeline. A further perspective lies in common principles of fungal virulence that may be executed by phyto- as well as human-pathogenic fungi and that have not been analysed on a comprehensive scale so far: understanding fungal pathogenesis in different hosts across kingdoms holds the promise of extracting fundamental modes of action that may be exploited for universal targets, thereby narrowing the scope of candidates when screening for novel antifungal compounds [38].

## Synthetic biology as a new technology for basic research and cell factories

Most strain development programs for industrially exploited filamentous fungi started by using classical mutagenesis and selection. This has been very successful in generating highly productive strains but has also led to strains that are often impaired in general fitness. This is mainly due to unfavorable mutations occurring and accumulating during consecutive rounds of classical mutagenesis. In addition, under certain conditions fungal strains are able to produce a huge number of secondary metabolites including mycotoxins and initiate developmental programs that interfere with industrial processes. To circumvent these pitfalls, the EUROFUNG consortium advocates a more targeted strain development approach eventually culminating in the design of an optimal fungal genome. Here synthetic biology tools in general and more specifically genome editing via CRISPR/Cas9 comes into play. The optimal fungal genome should contain all genes necessary for fitness, high productivity, and robustness in industrial conditions and has been gradually down-sized by eliminating dispensable metabolic pathways or secondary metabolite gene clusters.

### Genome editing and the CRISPR/Cas9 revolution

Over the past decade, the *ku70/ku80* tool box has made it faster and easier to study gene function in filamentous fungi. Despite this major improvement of gene targeting efficiency, only a full gene knock-out library for *Neurospora crassa* has been completed [39], and a second collection of whole genome gene knock-out mutants in *A. fumigatus* is on its way [40]. For *A. nidulans* a near complete set of deletion constructs was produced for each gene and is available for the community [41]. Despite the usefulness of high efficiently gene targeting by inhibiting non-homologous end joining, and robotizing the methods for constructing gene replacements cassettes, mutant generation and verification remains laborious. In addition, no genome-wide libraries exist for systematic GFP-tagging, gene overexpression or tunable expression of essential genes. The use of CRISPR/Cas9 systems for gene editing and loss of function mutants has proven to be a powerful tool for loss-of-function analysis [35, 42, 43]. Generating increase knowledge of gene function in filamentous fungi is now highly desirable to allow the development of robust cell factories and identification of targets to fight human and plant pathogenic fungi.

With the simultaneous expression of multiple guide RNAs, the CRISPR/Cas9 system allows multiplexed genome editing, offering the possibility to quickly create multiple gene knock outs in a single experiment. The multiplex CRISPR/Cas9 technology, in combination with marker free disruption of genes, make approaches to optimise cell factories feasible, for example in the production of secondary metabolites by removing all unwanted secondary metabolite gene clusters. Likewise, optimal cell factories for production of (recombinant) proteins will benefit from the simultaneous deletion of genes encoding extracellular proteases. However, parallel analysis of large numbers of mutants and the risk of cross contamination due to spore dispersal still remain a challenge and limit the speed of high-throughput screening for filamentous fungi. Hence, better methodologies need to be developed to culture filamentous fungi in microtiter plates with reproducible submerged growth characteristics and no or reduced sporulation.

CRISPR/Cas9 is not limited to the construction of gene disruptants: it can also be used for sophisticated genome modifications to create GFP-gene fusions to study protein localizations though exogenously supplied ‘donor templates’ that can be targeted to the gene of interested via a properly designed sgRNA [42]. Similarly, CRISPR/Cas9 allows the introduction of specific mutations, stop codons or alterations in potential transcription factor binding sites to study protein function or DNA–protein interactions. Finally, the CRISPR/Cas9 technology has great potential to study the function of essential genes and to study genes by overexpression by swapping endogenous promoters for tight, tunable and metabolism-independent promoter such as the Tet-On/Tet-Off system [44]. Deactivated Cas9 (dCas9) lacking endonuclease activity while maintaining an ability to specifically bind to DNA sequences can act as activator or repressor of target genes. This approach has been successfully used in *E. coli* [45, 46] and eukaryotes [47] and has great potential to prevent or activate gene expression in filamentous fungi.

### Synthetic chromosomes and genomes as future drivers of basic and applied sciences

A synthetic variant of the bacterium *Mycoplasma mycoides* has recently been constructed demonstrating life functions, including self-replication [48]. Similar efforts are ongoing for the yeast *Saccharomyces cerevisiae* [49]. These landmark advances offer the possibility of creating organisms that are tailored to support specific production processes or to answer specific research questions. As DNA synthesis and engineering tools are now ready to construct small genomes, it is intriguing to speculate that similar constructions will be possible in filamentous fungi in the near future. As for *M. mycoides* and *S. cerevisiae* [50, 51], one of the first goals is to define a minimal genome for filamentous fungi. Bioinformatic analyses of the many fungal genomes that are currently accumulating are likely to identify large numbers of dispensable genes that can be eliminated using Cas9 multiplexing gene deletion strategies. Such a chassis can be used as the clean background for the production of enzymes and secondary metabolites minimizing the risk of co-production of undesirable mycotoxins or undesired cross-chemistry that complicates pathway analysis and reduces yields. In addition, a fungus with a minimal genome will be an excellent host for analyzing multiple areas from aspects of the primary metabolism to development. A subsequent step could be *de novo* chromosome design implementing large expression platforms for multi-gene pathways for the production of metabolites requiring large gene clusters. Such platforms should be compatible with modular cloning strategies that efficiently combine elements such as promoters, terminators, selectable markers, and sequences that target fragments to defined expression sites, facilitating efficient strain construction. Insertion of large gene clusters may be performed by the combined use of intra-cellular homologous recombination to fuse PCR fragments with relevant genes and for insertion into the genome in a process that can likely be stimulated by Cas9 as has been shown for *S. cerevisiae* [52]. Potentially, platforms could be constructed that allow for insertion of many identical gene copies in a stable and defined manner to increase production of specific proteins. In the yeast Sc2.0 project, symmetrical loxP sites are inserted between non-essential genes and recombination between the sites can be induced by expression of the Cre recombinase [53]. Implementing a similar system in filamentous fungi may have a wide impact, considering that many fungi are difficult to cross as a sexual cycle does not exist, is elaborate and difficult to perform, or remains to be discovered. Uncovering the sexual cycle, or restoring it in industrial strains where is has been lost during strain development, has significant potential for strain development (e.g. [54]). Additionally, enhanced and defined recombination in a parasexual cycle based procedure could set the stage for efficient strain development. For example, haploid strains that have been selected after random chemical or radiation induced mutagenesis could be fused to form diploids. The genomes could then be scrambled by synthetic biology based recombination followed by haploidization to produce strains with new gene combinations that may further benefit production. This strategy may even be used for quantitative loci analyses to pinpoint genes that are relevant for desirable phenotypes [55].

## What are the optimal approaches to deposit and analyze fungal–omics data and to extract meaningful biological information from it?

Systems biology approaches have become integrated in nearly every research area involving fungi and one can hardly find an academic research project that does not leverage high-throughput technologies to analyses genomes, transcriptomes or proteomes. However, the EUROFUNG consortium foresees that this development also presents the wider research community with a number of key challenges.

### Quality of fungal genome datasets

Quality of fungal genome datasets: The participants agreed that the quality of fungal genome sequence data is highly variable, thus limiting the reliability of genome comparisons. While the limitation in quality of the early genome sequences was mainly due to the sequencing technology, more recent genome sequences—while having a higher quality and better assembly—suffer from poor annotation and propagation of errors due to automated annotation. Up to around 2010, a fungal genome sequence could be published in high impact journals, and therefore consortia were formed to improve the automated gene calling and annotation by manual curation and verification of genes [56, 57]. Nowadays, with the exponential increase in genome sequencing, very few genomes make it past the initial draft annotation and while these are better in terms of coverage and assembly, they generally lack well-supported and verified annotations. The use of automated annotation is dependent on existing annotated genomes as a reference, consequently any errors are likely to be propagated; an issue compounded by limited resources for manual annotation efforts. 

A key determinant of the utility of a genome sequences is the accuracy of gene calling. This is more complex in filamentous fungi than many organisms due to high numbers of introns and the prevalence of antisense transcripts. RNAseq has a major role in refining gene models but many studies are technically limited, for example employing strand-specific analysis is essential in understanding splicing frequency and antisense identity and prevalence and a wide range of growth conditions are required to observe a large proportion of transcripts. A fundamental aspect is the ability to define precisely the 5′ and 3′ ends of transcripts, as this helps identifying features such as the promoters, terminators and coding sequences. If miss-called, downstream experimental analysis, such as the production of mutant libraries for altered expression or protein tagging will be severely handicapped. Therefore, there is a need for the use of more refined techniques to define transcripts and genes, such as targeted analysis of both the 5′ and 3′ ends [58] as well as full-length transcript sequencing using technologies such as PacBio [59].

### Accessibility to fungal datasets

Currently, fungal genome sequences are hosted at different resources, such as JGI’s Mycocosm, AspGD, FungiDB, NCBI, and several others [60]. The groups generating the data often use different algorithms for assembly and annotation which can complicate the comparison of genomes that are not in the same resource. Furthermore, even though some genomes are included in all these resources, it is not always the same version of the annotation or sequence that is used and the tools to access them often use different default settings, which means that the ‘same’ search applied to the ‘same’ genome at a different resource can provide different results. This severely compromises the quality of fungal research and the reliability of the data generated, in particular by third parties. It is critical for the future of genomics-driven fungal research that anyone can have full access to reliable genomic information, and every effort should be made to make it so.

### Comparability of fungal datasets

For most fungal species only a single genome sequence is available. The choice of strain used for genome sequencing is often arbitrary: availability in the lab, typical model strain, type strain, etc. Even for type strains, this is in no way a guarantee that the selected strain is the most representative for a given species. For instance, a strain that has been in the lab for many generations may have lost some of its ‘natural’ characteristics such as virulence. The first isolate to be discovered in a species is often made the type strain, but may not be representative of the species complex. Therefore, conclusions about differences between species based on the genome of a single strain may not reflect the actual diversity of these species. Consequently, care should be taken when analysing such data.

Unfortunately, omics data resulting from these studies are rarely used in follow-up or comparative studies. There are two main reasons for this: First, omics data is typically deposited in raw formats in databases, which makes it de facto unusable for a large proportion of researchers who do not have the required bioinformatics skill set. Second, there is substantial variation in experimental conditions between datasets, which makes comparability difficult. While it will be hard to prevent this, it would be desirable for the research community to define ways to enable more comparative studies of datasets from different labs. Further challenges exist for fungal metabolomics, such as the absence of good fungal metabolite reference libraries, variation of methodology (e.g. different mass spectrometry platforms) and the absence of a good public repository of these data. As mentioned before, metabolomics has the potential to initiate new and improve existing applications but significant time and financial investments are needed to make this a reality.

### Maintenance and sustainable use of fungal datasets

Overarching all of the issues above is the availability and use of high-quality manually curated omics databases where genomes and other omics-studies can be shared. The participants of the meeting agreed that the effort required to maintain such resources, where literature and data is extensively curated by experts, is substantial but justified considering the added value which would come from such efforts. More accessible data, high-quality annotation, and a systematic framework to link research publications with the fungal genomes would significantly accelerate fungal research. Importantly, linking research findings to the respective genomes through public databases inevitably increases the impact of individual studies, the genomes and associated datasets. As the mass of fungal literature currently doubles every 7–8 years, sifting through the respective data and publications will only become more challenging in the future. Most importantly, European researchers, funding agencies and policymakers should seriously consider the way fungal genomics data should be organized and the European role in this. Currently, infrastructure for fungal genomics is strongly dependent on US and UK initiatives and very few European funded initiatives contribute to a well-organized infrastructure to host and analyze fungal genomes, transcriptomes and proteomes. Should access to the US infrastructures become limited for non-US scientists or US investment in databases significantly reduced, as recently seen for AspGD and the Broad Institute resources, this will create serious problems for European fungal research. Omics data is very cost effective but its utility is largely dependent on making the data accessible and usable by the wider research community. Without suitable investment in appropriate resources and structures much of the potential value of such data is lost and the investment in the original studies squandered. Most alarmingly, the National Human Genome Research Institute (NHGRI), which funds several of the model organisms’ databases (yeast, worm, fly, zebrafish, and mouse) and the Gene Ontology consortium, has recently announced there will be decreased funding by 30 % over the next few years for these resources [61, 62].

## What are fungal model organisms good for?

Both the useful facets of filamentous fungi and in some cases their devastating biological activities are intimately rooted within the ‘filamentous mode of life’. The ‘reference system’ for filamentous fungi has been, traditionally, the budding yeast *S. cerevisiae*, due to its phylogenetic relatedness to filamentous fungal ascomycetes and to the wealth of knowledge colleagues working with this unicellular ascomycete have amassed. However, it must be acknowledged that the budding yeast is, for many aspects, too specialized and limited to play the same lighthouse role in the future. To put just a few examples, the genomic duplication that took place in the Saccharomycotina line [63] resulted in a notable degree of gene redundancy, which often complicates functional analyses. Moreover, even with the genome duplication notwithstanding, the budding yeast genome codes for half the number of genes of filamentous ascomycetes. As many fungal genes do not have a yeast orthologue a large proportion remain ‘uncharacterized’. Accurate annotation of filamentous fungal genomes had to overcome the difficulties imposed by the abundant introns, a problem that was not encountered by our budding yeast colleagues. Budding yeast and its ancestors have lost RNA interference mediated transposon resistance and exhibit a high internal reverse transcriptase activity combined with an efficient homologous recombination system which is presumed to account for the unusually low number of introns [64]. The *S. cerevisiae* network of metabolic pathways, streamlined for growth on ‘compositionally specialized’ substrates, is very limited in the case of primary metabolism, and in the case of secondary metabolism essentially absent. This makes the ‘model’ yeast a poor model for studies aimed at understanding and exploiting many biotechnologically useful features of fungal metabolism, including the often neglected cell biology of secondary metabolite production. In terms of basic cell organization, laboratory strains of budding yeast have usually lost the natural ability to grow as filaments, which is restricted to pseudohyphal growth [65]. Remarkably, budding yeast uses microtubules (MT) solely for mitosis and nuclear positioning, whereas the organization of filamentous fungal hyphae largely relies on MT dependent transport, such that MT-dependent processes strongly affect growth, including invasiveness and pathogenicity, as well as secondary metabolite and enzyme production. From the organismal point of view, filamentous fungi undergo developmental processes, shaping the sexual and asexual reproductive structures that have no parallel in the budding yeast. As development appears to be intimately connected to secondary metabolism [66] and, undoubtedly, to fungal pathogenicity - there is no infection without efficient propagation. For these and many other reasons we need to turn our gaze to organisms other than *S. cerevisiae*. Thus it seems clear that future progress will be less yeast-centric, emphasizing the need for better fungal experimental ‘models’.

The term ‘model/s’ can be misleading, and requires a shift in focus towards the more appropriate term ‘reference/s’ organism/s, more suited to match specific problems. For example, it is hardly disputable that the ‘reference’ species to understand conidiophore development, or to unveil fundamental differences between MT-mediated intracellular traffic between filamentous ascomycetes and the budding yeast is *A. nidulans* [67], but arguably no sensible colleague would try to understand citric acid production without metabolic models based on *A. niger* as ‘reference’, or will work on the Spitzenkörper without turning his/her eyes to studies with *N. crassa* [68]. This discussion on ‘references’ boils down to a reconsideration of the ‘basic unit of research’, which arguably should be ‘the problem’ (*i.e.* ‘germination’, ‘dimorphism’, ‘sensing’) rather than ‘the organism’. Indeed the mechanistic details may be slightly different in the different instances (i.e. *organisms*), and such a ‘problem-centric’ approach might be helpful to sort out the common denominator (the actual basic-thus common-gear). This problem-focused approach might enable a more holistic understanding of the fungal kingdom.

A frequently encountered problem common to all species is the need for better tools for gene inactivation. Studies with knockout alleles, albeit useful to take a quick glance at the function of a given gene, is restricted to those genes that are not essential or nearly so (a score of knockout alleles debilitate growth to an extent that although not strictly leading to lethality preclude any further study). A way around the ‘essentiality problem’ is the use of tight conditional expression systems [44]. Null mutations only provide clear mechanistic insights, when e.g. a gene encoded enzyme activity can be measured or a linear signal transduction pathway is interrupted. However, the mechanistic conclusions are highly limited, when phenotypic consequences reflect ‘collateral damage’ rather than the absence of the specific function of the gene in question. Here, conditional expression systems are limited by the fact that the turnover of the protein whose function is inactivated might be unbearably long. Thus, there is an urgent need of tools that permit the acute inactivation of gene expression. Thus far, such weaponry is reduced to temperature-sensitive alleles, which ideally inactivate a protein only in cells incubated at the ‘restrictive’, but not at the ‘permissive’, temperature, usually by promoting protein misfolding/degradation. One problem is that these mutations are available largely in those organisms that have been traditionally used for classical genetic studies, such as *N. crassa*, *A. nidulans* and *Ustilago maydis,* but scarce in industrial or clinical species. A second is that temperature-sensitive growth does not necessarily reflect the complete inactivation of an otherwise essential protein at the restrictive temperature, but in many cases, a stronger requirement for the function of the said protein at elevated (stress-causing) temperatures, under which a hypomorphic allele will not sustain growth. A third is that if the restrictive temperature for a given allele is too high, its usefulness may be limited (for example, it may not be suited for in vivo microscopy studies, as the optics cannot be ‘heated’ beyond 38 °C). Thus there is a very strong need for better genetic switches, such as those based on conditional degrons inspired in tools developed for the budding yeast [69]. Doubtless, the development of these and other tools for conditional gene inactivation will be strongly influenced by the recent development of CRISPR/Cas9 technologies for filamentous fungi as discussed above. Note that CRISPR will not only deeply impact experimentation but legal regulations as well [70].

Lastly, there is a strong need for ‘reference’ organisms to be amenable to classical genetics, the feature that in the first place made filamentous fungi useful experimental systems. For example, the identification of causative mutations by genomic mapping is largely facilitated by previously knowing the localization of the mutation in a genetic map [71, 72]. Also transformation is mutagenic and it should be a rule of thumb to demonstrate that the phenotype of a recombinant allele segregates in a Mendelian fashion, as otherwise the phenotype might reflect a synthetic effect of more than one mutation (i.e. the ‘designed mutation’ plus extragenic transformation-induced mutations that improve the fitness of the single mutant [73]). Finally, many important fungal characteristics will be polygenic and their understanding will require experimental systems permitting those mutant versions of many genes are combinable with relative ease by meiotic recombination.

## Future important technologies for filamentous fungi

Fungi are efficient enzyme producers but their relative inability to make heterologous proteins limits their usefulness. A key issue is that our understanding of the different pathways of exocytosis is very limited, and actually we do not even know how many ways a cargo has to reach the extracellular milieu. A major standing bottleneck is the detailed understanding of these pathways and their regulatory circuitry to allow their tailored genetic manipulation. A better comprehension of intracellular traffic may also ‘permeate’ to secondary metabolite production. For example, the final steps of penicillin biosynthesis take place in the peroxisomes, such that these organelles have a profound influence on antibiotic titers [74]. Lastly, hyphal growth is strictly dependent on exocytosis, and thus essential fungal-specific aspects of intracellular traffic would be druggable for antifungal intervention. A key issue is that with the notable exception of the unfolded protein response [75], intracellular traffic is mainly regulated at post-transcriptional levels by small GTPases, lipids and their respective effectors [76]. Several technological developments will be helpful to gain further insight.

(i) *In vivo imaging:* Studies with fungal cells growing on the microscopy stage using movies with high time resolution. On the cell side these studies will require brighter fluorescent markers, ideally fully functional fluorescent fusion proteins expressed at physiological levels and, from the hardware side, equipment permitting 5-way multidimensional acquisition (x, y, z, time, multiple channels) of images. These studies should be combined with acute inactivation of gene function ‘on-the-stage’, for example by exploiting available heat-sensitive mutations [77]. Besides suitable microscopy hardware these studies will demand expert know-how on the techniques to image cells without perturbing the process that is being observed. Moreover, improving the time resolution of time-lapse sequences implies progressively larger files that demand specialized software, dedicated high-capacity digital storage resources and powerful computer workstations, a triple combination that will be only available in highly specialized central facilities. Lastly, the future of these microscopy studies undoubtedly passes through systems biology, and our capacity to perform automated image analysis, to statistically interpret large amounts of data and to match them to mathematical models.
(ii) *Biochemistry combined with shotgun proteomics:* Intracellular compartments are transient entities, and thus difficult to define unless their composition is very precisely determined. We need better subcellular fractionation procedures to obtain homogeneous populations of organelles and connecting carriers, possibly aided by appropriate genetic blocks. The composition of these organelle fractions needs to be elucidated by shotgun proteomics and by lipidomics (a technology that is not generally available). Proteomics is often limited by the quality of the reference databases because genes/proteins are miss-predicted by automated gene call procedures. Improving the quality of peptide reference databases is essential to obtaining more reliable proteomics data. This objective should be a major target of genome information repositories.
(iii) *Electron microscopy:* The subcellular organization of filamentous fungi will not be understood without exploiting the recent developments in this field. Whenever used by technically competent laboratories tomography studies have yielded significant insights (see for example [78]). Further EM studies should undoubtedly involve the largely unexploited capabilities of immune-EM (for example see [79]) and correlative EM-light microscopy [80].
(iv) *Other technologies:* Three further technologies were identified by the EUROFUNG group to be of crucial importance for basic and applied filamentous fungal research. On the one hand, single-cell analytics will offer tremendous opportunities to study population heterogeneity at both the biological level (stochastic variations, molecular noise, regulatory effects) and environmental level (inhomogeneity, fluctuations and gradients in natural habitats and bioreactors). Microfluidic single-cell cultivation devices equipped with time lapse imaging for high-throughput omics analyses are available for academic groups studying unicellular microorganisms [81] but not yet for filamentous fungal systems. There is thus a need for input from these research communities combined to make these technologies widely applicable for filamentous fungi [82]. Another technology frequently employed with unicellular microorganisms but that is underdeveloped for filamentous fungi is flow cytometry. This technique in combination with cell sorting is very powerful for high-throughput screenings, for example. Some preliminary approaches have already been published using large-particle flow cytometry [83, 84]; however, further developments are clearly necessary. A third potentially important technological development has been the application and refinement of single-use bioreactors, such as wave-mixed bioreactors, for the medium-scale production of high-value products (up to 2.5 m^3^). These offer many opportunities for filamentous fungi as cell factories in pharmaceutical and cosmetics industries. Not only is this platform technology safer (decreased risk of microbial contamination and cross-contamination), greener (reduced requirements for cleaning and sterilization), faster and more flexible (easy process and product change), cheaper (saving of time and costs) and smaller (reduced facility footprint) [85], it offers better oxygen transfer rates, more homogeneous energy dissipation and comparable or better growth of filamentous fungi when compared to their cultivation classical stirred tank reactors [85, 86]. Of special interest for small-scale controlled solid state fungal (co-)cultivations (max. 25 L) are reusable gas–liquid-solid bioreactors based on the membrane gradostat bioreactor technology. They provide a low shear environment, do not need the addition of antifoam agents and give similar or better results than in stirred reusable bioreactors as shown for *A. niger, N. crassa* and *Penicillium* spp. [87].

## Which training programs are key for the next-generation of fungal scientists?

There is a shortage of skilled researchers with multidisciplinary expertise in fungal molecular biology and biotechnology. The participants agreed that advanced research on fungal biology and biotechnology requires a multi-scale view—i.e. the combination of knowledge on cellular and molecular biology with mathematical modeling, statistical/computational tools and industrial production—and hence a high level of expertise in these disciplines. Moreover, transfer of in silico and experimental skills between the complimentary areas of medical mycology, plant pathology, and industrial microbiology are severely limited. Hence, training of multidisciplinary scientists with strong background in all fields will create an unparalleled environment for future developments such as antifungal drug discovery and new technological applications of filamentous fungi. In addition, industrial partners of EUROFUNG identified that there is a lack of staff within the biotechnology industry with expertise in fungal systems—consequently bacterial and yeast systems tend to be the default, when fungal systems may be the rational choice. This underlines the need for better training, particularly at the postgraduate levels and improved mechanism to promote knowledge transfer if we are to realise the potential that fungal biology offers.

There was firm agreement that ‘technician level bioinformaticians’ are needed. These are trained bioinformaticians (e.g. to a bachelor or masters level) who are not actively involved in hypothesis generation, but fill an essential role by conducting bioinfomatic experiments and curating resources, rather than developing and testing new software. This would increase lab bioinformatics capacity and enable the exploitation of growing in silico resources.

Finally, there is a barrier for knowledge exchange between industry and academia. Companies have become repositories of key knowledge and innovative technologies needed for education and research but the dissemination of this knowledge is limited due to the lack of coordinated training between the two sectors. Likewise, much academic research does not reach relevant industrial research and business development activities. Hence, future multidisciplinary training programs should involve cross-sectoral elements to enable the new generation of fungal scientists and biotechnologists to understand the wider and economical potential of their work.

## Conclusions

Significantly improved scientific understanding and better translation of knowledge to underpin innovation is required to improve filamentous fungal based cell factories and to better control pathogenic fungi. Crucial for this are continuous efforts for high-quality annotation and curation of fungal genomes, with continuous financial support to make omics data and resources publicly available and comparable. Furthermore, a sophisticated synthetic biology tool box tailored to a range of filamentous fungal species needs to be developed to overcome our limited flexibility to genetically modify and optimize them. Learning from work with well-chosen reference organisms, integrating systems-level functional studies of pathogen and industrial organism biology will facilitate the development of new products, biosynthetic systems and new anti-fungal drug discovery programmes. The fungal biotechnology revolution is happening—the more young academics become trained and interested in multidisciplinary fungal research the better! With their passion and enthusiasm the huge potential of filamentous fungal systems will be faster and better exploited, which will help society to address key challenges of the twenty-first century.

## Appendix: Participants of the meeting

Mikael Andersen, DTU Copenhagen

Sharief Barends, Dupont

Gerhard Braus, Göttingen University

Hans van den Brink, Chr. Hansen

Mark Caddick, University of Liverpool

Timothy Cairns, TU Berlin

David Canovas, University of Seville

Luis Corrochano, University of Seville

Ronald de Vries, CBS Utrecht

Dieter Eibl, ZHAW Wädenswill

Regine Eibl, ZHAW Wädenswill

Craig Faulds, Polytech Marseille

ErzsébetFekete, University of Debrecen

Thomas Haarmann, AB Enzymes

Kim Hansen, Novozymes

Ingo Hartung, Bayer Pharma

Ritchie Head, Ceratium

Christiane Hertz-Fowler, Fungi DB

Levente Karaffa, University of Debrecen

Frank Kempken, Kiel University

Jochen Kleemann, Bayer Crop Science

Rudibert King, TU Berlin

Dietrich Kohlheyer, Forschungszentrum Jülich

Sven Krappmann, University Erlangen-Nürnberg

Nada Krasevec, Ljubljana University

Uffe Mortensen, DTU Copenhagen

Vera Meyer, TU Berlin

Jolanda van Münster, University of Nottingham

Igor Nikolaev, DuPont

Miguel Peñalva, CSIC Madrid

Arthur Ram, Leiden University

Monika Schmoll, Austrian Institute of Technology

Bernhard Seiboth, TU Vienna

Oksana Shegay, Puratos

Klaus Tietjen, Bayer Crop Science
